# Effect of Cellular Quiescence on the Success of Targeted CML Therapy

**DOI:** 10.1371/journal.pone.0000990

**Published:** 2007-10-03

**Authors:** Natalia L. Komarova, Dominik Wodarz

**Affiliations:** 1 Department of Mathematics, University of California Irvine, Irvine, California, United States of America; 2 Department of Ecology and Evolution, University of California Irvine, Irvine, California, United States of America; Institute for Medical Biomathematics, Israel

## Abstract

**Background:**

Similar to tissue stem cells, primitive tumor cells in chronic myelogenous leukemia have been observed to undergo quiescence; that is, the cells can temporarily stop dividing. Using mathematical models, we investigate the effect of cellular quiescence on the outcome of therapy with targeted small molecule inhibitors.

**Methods and Results:**

According to the models, the initiation of treatment can result in different patterns of tumor cell decline: a biphasic decline, a one-phase decline, and a reverse biphasic decline. A biphasic decline involves a fast initial phase (which roughly corresponds to the eradication of cycling cells by the drug), followed by a second and slower phase of exponential decline (corresponding to awakening and death of quiescent cells), which helps explain clinical data. We define the time when the switch to the second phase occurs, and identify parameters that determine whether therapy can drive the tumor extinct in a reasonable period of time or not. We further ask how cellular quiescence affects the evolution of drug resistance. We find that it has no effect on the probability that resistant mutants exist before therapy if treatment occurs with a single drug, but that quiescence increases the probability of having resistant mutants if patients are treated with a combination of two or more drugs with different targets. Interestingly, while quiescence prolongs the time until therapy reduces the number of cells to low levels or extinction, the therapy phase is irrelevant for the evolution of drug resistant mutants. If treatment fails as a result of resistance, the mutants will have evolved during the tumor growth phase, before the start of therapy. Thus, prevention of resistance is not promoted by reducing the quiescent cell population during therapy (e.g., by a combination of cell activation and drug-mediated killing).

**Conclusions:**

The mathematical models provide insights into the effect of quiescence on the basic kinetics of the response to targeted treatment of CML. They identify determinants of success in the absence of drug resistant mutants, and elucidate how quiescence influences the emergence of drug resistant mutants.

## Introduction

Cellular quiescence is a central process that regulates the kinetics of cellular proliferation and tissue homeostasis, especially in stem cells [1]–[6]. If stem cells are not needed to divide and to replenish tissue cells, they temporarily stop to progress through the cell cycle until further divisions are required. Several cancers are thought to be maintained by “cancer stem cells” in a similar manner as healthy tissue is maintained by regular stem cells [7]–[10]. That is, the primitive cells divide and give rise to cells that are differentiated to a certain degree, at least during the earlier stages of the disease. Cancer stem cells are thought to be an important target for any therapy that aims to eradicate the tumor [11], [12] . If the stem cells are not eliminated, the cancer is likely to relapse [13]. While primitive cancer cells proliferate with a higher rate than healthy cells, data indicate that they can still undergo quiescence, both during tumor growth and during treatment . An example of where this has been demonstrated is chronic myelognous leukemia (CML) [14], [15]. It is even possible that in this case, therapy induces quiescence in primitive cancer cells [16]. Quiescent cells in turn are not affected by the drug and are therefore shielded from therapy-induced elimination [16].

Chronic myelogenous leukemia (CML) is a cancer of the hematopoietic system which progresses in three phases: the chronic phase, the accelerated phase, and blast crisis [17]–[20]. It is thought that cell growth is brought about by the proliferation of cancerous stem cells and progenitor cells [21]. During the chronic phase of the disease, the fraction of immature cells is relatively low, while a sharp increase in the fraction of immature cells is observed as the disease progresses. It is thought that CML initiation and progression is driven by the product of the BCR-ABL fusion gene (Philadelphia chromosome) [17]. The BCR-ABL protein has a constitutively activated tyrosine kinase, activating multiple signal transduction pathways. This leads to excessive cellular proliferation, reduced apoptosis, and decreased cellular adhesion. Imatinib mesylate (STI571 or Gleevec) is a targeted inhibitor of the BCR-ABL fusion protein and has given rise to impressive treatment results, especially when treatment is started during the chroninc phase of the disease [20], [22]–[28]. Blood cell counts return to normal levels, and the levels of the BCR-ABL gene can even become undetectable. While patients tend to relapse after cessation of Imatinib treatment [29]–[32], a recent study has shown that some patients did not show any relapse as long as two years after treatment cessation, raising the possibility that CML has been eradicated from these patients [33].

There are two major barriers to CML eradication by Imatinib. First, not all cells in the heterogeneous CML population are equally susceptible to treatment. The problem lies especially with the population of stem cells. While some have argued that stem cells are not at all susceptible to Imatinib [30], another scenario is that stem cells are susceptible to treatment while in an active state, but are not affected by the drugs while in a quiescent state [13], [16], [29]. Because primitive CML cells have been observed to undergo quiescence, and because Imatinib itself might trigger a quiescent state in some cells, tumor eradication can be a difficult task. Second, the tumor cells can evolve acquired resistance to Imatinib [22], [23], [25], [27], [28], [34]–[40]. This can be conferred by point mutations or gene amplification events. The probability that a resistant cell is generated in turn depends on the growth kinetics of the cancer cell population, which are influenced by regulatory processes such as quiescence and cell death.

This paper investigates how cellular quiescence influences the kinetics of the treatment response, and the probability of treatment failure as a result of acquired resistance. Initiation of therapy can result in three patterns of tumor cell decline in the model. In one parameter region, we first observe a fast phase of tumor cell decline (roughly corresponding to the eradication of cycling cells by the drug), followed by a slower phase (awakening and death of quiescent cells), a pattern which has been observed in clinical data [29], [30]. For this case, we define mathematically the time when the switch to the second and slower phase occurs. The other two patterns of tumor cell decline are a one-phase decline and a reverse biphasic decline. Eventually, the model predicts the extinction of the CML cells, and defines the time when extinction occurs. Depending on the parameter values, this may or may not occur in a realistic period of time. The calculations therefore define conditions under which imatinib therapy fails to eradicate the cancer, and when eradication can be successful. A more elaborate model includes the ability of tumor cells to acquire mutations that confer drug resistance. We find that in the context of treatment with a single drug, parameters that determine the kinetics of cellular quiescence do not affect the probability of treatment failure as a result of drug resistant mutants. On the other hand, if two or more drugs are used in combination to treat the cancer, then treatment failure as a result of drug resistance is promoted by the occurrence of cellular quiescence. Interestingly, while cellular quiescence significantly prolongs the time until the cancer has dropped to low numbers or has been driven extinct, the model predicts that drug resistance does not evolve during this treatment phase in this case. Increased cellular quiescence increases the likelihood that resistant mutants are generated during the growth phase of the cancer before therapy is initiated.

## Results

### Treatment in the absence of drug resistant mutant cells

We formulate a stochastic model that includes a population of primitive, proliferating CML cells, and a population of quiescent CML cells. The proliferating cells divide with a rate *l* and die with a rate *d*. The death rate captures both the natural death rate of cancer cells and the treatment-induced death rate. In the absence of treatment, *l>d*, and the cell population grows exponentially. Treatment increases the parameter *d*. If treatment is efficient, then *l<d*, such that the tumor cell population declines. The cells enter a quiescent state with a rate *α*, and quiescent cells re-enter the cell cycle with a rate *β*. Note that quiescent cells do not divide or die and are not susceptible to any drug activity. We first consider the average numbers of proliferating and quiescent cells, *x(t)* and *y(t),* as a function of time. This can be described by the following pair of ordinary differential equations:
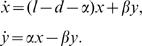
Note, that this model does not explicitly take into account differentiated CML cells. These are not thought to contribute significantly to malignant growth and are simply proportional to the number of primitive CML cells.

**Figure 1:**
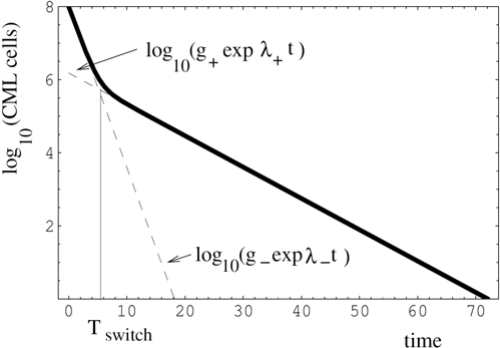
Biphasic decline of the CML cell population as a function of time, for parameters *l = 1*, *d = 1.5*, *α = 0.01*, *β = 0.2*, *I_0_ = 10^8^* and *J_0_ = 10^2^*. The solid line represents *log_10_(x(t)+y(t))*, and the dashed lines are *log_10_(g_+_exp{λ_+_t})* and *log_10_(g_-_exp{λ_-_t})* (See Text S1, Section 1.1 and 1.2 for details). The time of treatment in this case is T_treat_ = 72.1 and the switching time is T_switch_ = 5.1.

Assume the existence of a number of primitive CML cells, a fraction of which is quiescent. They are treated with the drug imatinib. In this first model, we assume that all CML cells are susceptible to the drug and that no drug resistance is generated by mutation. In this scenario, the death rate of the CML cells is greater than their division rate (*d>l*), such that the population of cells declines. The model suggests various behaviors upon initiation of treatment. In one parameter region, therapy results in two distinct phases of exponential decline (Figure 1), as observed in experimental data [29], [30]. First, the population of cells declines exponentially with a relatively fast rate, λ_-_, as a result of the death of proliferating cells, *x*. Then, a slower phase of exponential decline at a rate λ_+_ is observed because the quiescent cells become dominant and are only killed when they wake up and re-enter a cycling state. The values *λ*_±_ are given by 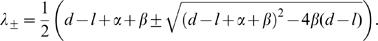 For small values of α the expressions for the decay rates simplify and we have *λ*_+_ *= -(d-l)* and *λ_-_ = −β* , that is, the first wave of decline happens at the net decay rate of cycling cells and the second wave happens at the rate of cell awakening.

In this model, treatment will eventually drive the tumor to extinction, but the time it takes to achieve this goal is influenced by the kinetics of the second, slower phase of decline, and thus by the rate at which cells enter the quiescent state, and the rate at which cells exit the quiescent state. The higher the rate at which cells enter quiescence, and the slower the rate at which cells exit quiescence, the longer it takes to reduce the CML population towards extinction. Also, the lower the overall death rate of cells, the longer it takes to reduce the tumor towards extinction. In the model, the time of the switch between the two phases of decline (Figure 1) is given proportional to 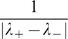, and the time of extinction is proportional to 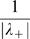 (see supplementary information, Text S1 Sections 1.2 and 1.3 for the exact expressions).

Note, however, that these dynamics are not universal in the model. This type of biphasic decline occurs if the death rate of cells is larger than the sum of the division and quiescence rates (*d>l+α+β*). For smaller death rates, when this condition is not fulfilled, two further patterns of decline are observed. Either the population of cells declines in a single exponential phase during treatment, or a first and slower phase of cell decline is followed by a second and faster phase of cell decline (a reverse biphasic decline). Exact mathematical conditions for these parameter regions are given in Text S1, Section 1.1. This behavior is observed if there is more quiescence in the population of tumor cells. In this case, the first phase need not be the fastest anymore, because it can be dictated by the kinetics of cell activation rather than cell death. Once a sufficient number of cells has been activated, cell death is the dominant factor and the rate of cell decline speeds up.

**Figure 2:**
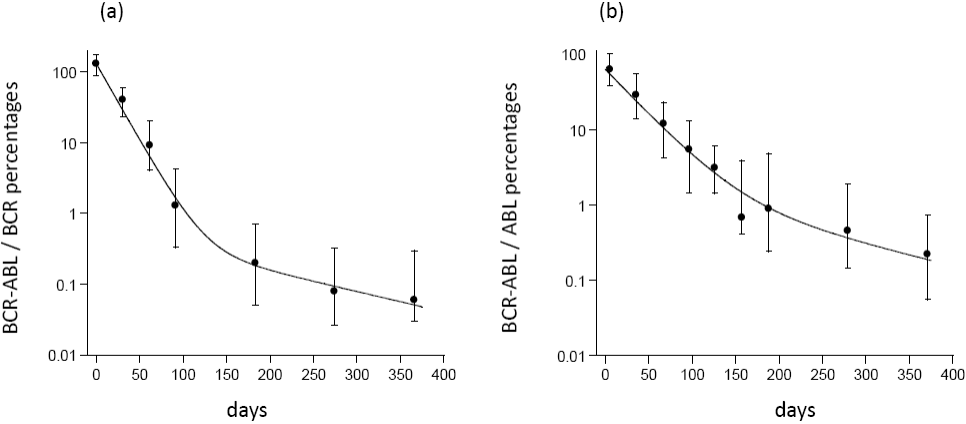
The relative amount of CML cells as a fuction of time, in patients treated with Imatinib. The circles represent experimental data replotted from (a) Michor et al [30] and (b) from Roeder et al [29]; they show the median values of BCR-ABL transcripts (relative to BCR transcripts in (a) and ABL transcripts in (b)). The vertical bars are the quartiles. The solid lines represent the fitted theoretical curves, formula (7) of Text S1, obtained by a mean-square procedure. The estimated parameter values are: (a) d-l = 0.0502 days^−1^, β = 0.0065 days^−1^, α = 10^−5^ days^−1^, J_0_ = 0.47; (b) d-l = 0.0278 days^−1^, β = 0.0067 days^−1^, α = 0.0004 days^−1^, J_0_ = 0.50. Here J_0_ denotes the initial percentage of quiescent CML cells.

**Figure 3:**
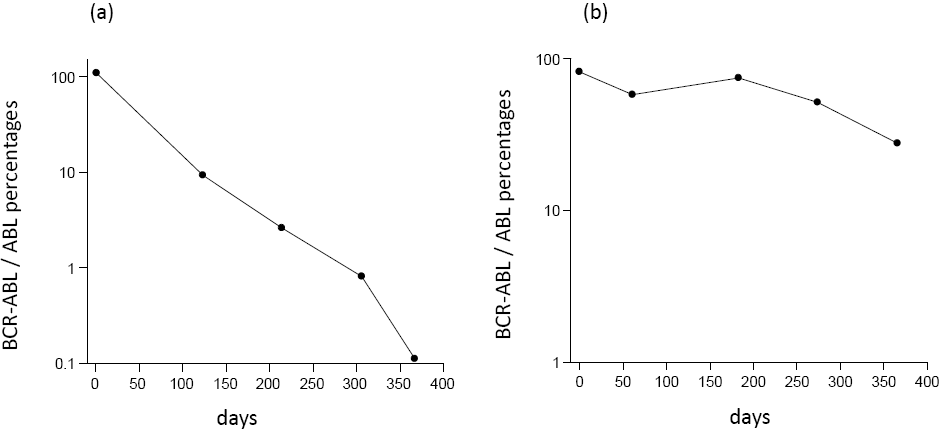
Data that document the decline of CML cells during imatinib treatment in two patients, taken from Roeder et al [29].

In order to show that our equations can accurately describe clinical data, we fitted the model to two data sets that document a bi-phasic decline of CML cells during treatment (Figure 2). Details of the data fitting procedures are given in Text S1, Section 1.4. The first data set is taken from Michor et al [30] and contains median BCR-ABL transcript levels from a selected cohort (n = 68) that excludes cases with transiently increasing BCR-ABL transcript levels (Figure 2a). The second data set is taken from Roeder et al [29] and contains median BCR-ABL transcript levels from an unselected cohort (n = 69) of CML patients (Figure 2b). In addition to the median values, Roeder et al presented individual responses to imatinib therapy. Figure 3 re-plots the clinical data from two patients that do not show a bi-phasic decline. Based on our model, it can be hypothesized that in these patients the number of CML cells declines in a single exponential phase during treatment (Figure 3a), or according to the reverse biphasic decline pattern (Figure 3b). However, analysis of additional data for longer periods of time will be necessary to test this hypothesis.

We can also investigate the dynamics of CML decline during treatment in stochastic terms rather than considering the average behavior of the population of CML cells. That is, assuming that we start with *I_0_* cycling cells and *J_0_* quiescent cells, we examine the probability that the population of CML cells is extinct. This probability increases monotonically with time and tends toward one as time goes to infinity. We can calculate the time when the probability of CML extinction approaches one. As expected, a higher rate at which cells enter quiescence and a lower rate at which cells exit quiescence increases the time until the probability of tumor extinction converges to one.

In summary, whether or not CML can be cured by imatinib therapy in the absence of acquired resistance depends on the time it takes for the cancer cells to be driven extinct by the treatment, and this in turn depends on the rate constants. Eventual CML extinction is the only theoretically possible outcome in the presence of therapy, but it may be achieved after a period of time that is longer than the life-span of the patient. Variations in parameters that determine the kinetics of cellular quiescence can determine whether relapse is observed in patients that stop imatinib treatment after a certain period of time [33]. Note that our notion of treatment induced “cancer extinction” is a mathematical one, that is, in the model we analyze here, the cancer cell population goes extinct, which corresponds to a cure. In patients, however, other complicating factors not included in this model may render tumor extinction a difficult goal to achieve by treatment. Therefore, our mathematical notion of “tumor extinction” should be translated into “clinical remission” in a medical context.

### Quiescence and the generation of drug resistant mutants

In the next, more complete model, CML cells can mutate to give rise to acquired drug resistance. In particular, we assume that during cell division, a resistant mutant is generated with a probability *u*. We further assume that CML cells grow exponentially to a defined size *N*, after which the disease is detected and imatinib therapy is started. We calculate the probability that the cancer is driven extinct by therapy, i.e. the probability that no resistant mutants spread before the CML cells have gone extinct. We examine how this probability depends on the parameters that determine cellular growth, mutations, quiescence and death. When talking about tumor extinction in the model, we always imply extinction brought about by drug therapy. As noted before, this should be thought of as “clinical remission” in medical rather than mathematical terms.

A previous model studied the probability of treatment failure as a result of drug resistance, but did not take into account cellular quiescence [41]. There, the result was obtained that the treatment phase is largely irrelevant for the generation of resistance. That is, if treatment does fail because of drug resistant mutants, these mutants were generated in the growth phase before the start of therapy. Quiescence can significantly slow down the rate with which the tumor cell population declines during treatment, thus prolonging this phase. The argument has been made that the tumor might acquire resistance during this phase and that this could lead to a relapse of the tumor after a certain time, despite continued therapy. We have performed a similar analysis with the current model, and found that even in the presence of quiescence, the treatment phase is not relevant for the generation of drug resistant mutants, no matter how long treatment takes. Thus, if at the start of therapy no resistant mutants exist, treatment is likely to result in the extinction of the tumor, given enough time (see Text S1 Section 2.2 for calculations).

**Figure 4:**
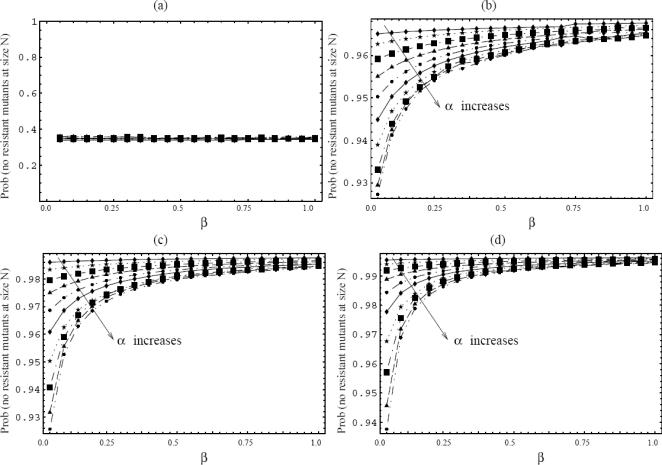
The probability of having no fully-resistant mutants at size *N* for different quiescence parameters. The numerical simulations are performed according to the theory described in Text S1, Section 2.3. Each figure (a)–(d) shows the probability of no resistant mutants as a function of *β* (the rate of cell awakening), for 10 different values of *α* (the rate at which cells become quiescent), α = 0.1, 0.2, … and 1.0. (a) Treatment with *m = 1* drugs; all the curves corresponding to different values of α are the same. The parameters are *N_0_ = 10^7^* and *u = 10^−7^*. (b) Treatment with *m = 2* drugs, *N = 10^11^*, *u = 10^−7^*. (c) *m = 3* drugs, *N = 10^13^*, *u = 10^−6^*. (d) *m = 4* drugs, *N = 10^13^*, *u = 10^−5^*. In all plots, we took *M_0_ = 10^3^*, *l = 1*, *d = 0*. The reason we used different values of *N* and *u* for different values of *m* is because we chose the parameter regime corresponding to intermediate values of the probability of treatment success. When this probability is nearly 100% or nearly 0, then the dependence on *α* and *β* is less apparent and less meaningful.

With this in mind, we calculate the probability of treatment success depending on the rate at which cells enter quiescence, *α*, and the rate at which cells exit the quiescent state, *β*. Several scenarios are considered. First we study resistance against a single drug (i.e. imatinib in CML treatment). We then also take into account resistance against 2 or more drugs used in combination. This is relevant because in addition to imatinib, further drugs are being developed that could be used in combination with imatinib to treat CML [23], [42]. In the main body of the paper we only present intuitive arguments. The rigorous calculations are given in Text S1 Section 2. Throughout the next few paragraphs we make the simplifying assumption that the cell death rate in the pre-treatment phase is zero. Also, the theoretical explanations will concentrate on one of the quiescence parameters, *α*, which is the rate of entering the state of quiescence. The rate of cell awakening, *β*, can be treated similarly (see e.g. Text S1, Section 3.4). Figure 4 illustrates the *α*- and *β*- dependence of the probability of no resistance. It was created by numerical solutions of ordinary differential equations for the characteristics, see the theory of Text S1, Section 2.3. The calculations give rise to the following findings.

#### Probability of one-drug treatment failure (due to resistance) is independent of quiescence

The probability to observe treatment failure as a result of resistance in the context of a single drug is not affected by quiescence parameters (Figure 4a). To put this in quantitative terms, the probability to have at least one resistant mutant at size *N* is independent of *α* and *β*.

**Figure 5:**
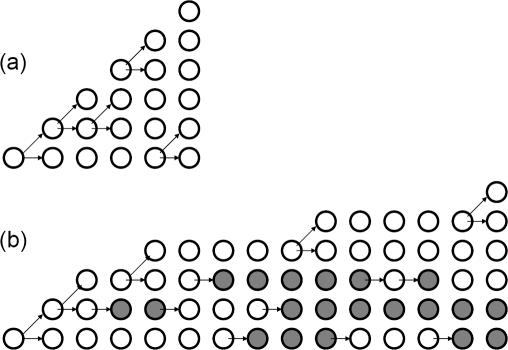
A schematic demonstrating the number of cell divisions that is needed for a colony of cells to expand from 1 cell to *N* cells (in the figure, *N = 6*). Empty circles represent cycling cells, and gray circles represent quiescent cells. Columns depict states of the colony in consecutive moments of time. The changes are marked by arrows. Two arrows stemming from one cell represent a cell division. A single arrow represents either a cell becoming quiescent or a quiescent cell waking up. (a) A colony without quiescence. (b) A colony with quiescence. In both cases we can see that it takes exactly N-1 = 5 cell divisions to expand to size *N*; however the process in (b) contains more “events”.

This is demonstrated by the following argument (see also Text S1, Sections 3.2 and 3.3) . Let us assume for simplicity that there is no cell death in the colony (all the arguments can be extended to nonzero death rates). In the model, mutants are generated during cell division. The probability of resistance is the same as the probability to generate mutants, which is defined by the number of cell divisions (and the constant mutation rate). It is easy to see that the total number of cell divisions until the tumor reaches size *N* does not depend on the quiescence parameters *α* and *β*. For instance, if there is no cell death, then the number of cell divisions to expand from one cell to *N* cells is exactly *N-1*, no matter what the quiescence rates are, see Figure 5. It is of course the case that the higher the rate at which cells enter quiescence, and the lower the rate at which cells exit quiescence, the longer it takes the tumor to grow to size *N*. However, the actual number of cell divisions to reach size *N* is unchanged by quiescence. Therefore, the probability to produce resistant mutants is independent of quiescence rates.

As we will see in the following paragraphs, the situation is different when considering resistance against two or more drugs. For treatment with multiple drugs, the probability of treatment failure as a result of resistance depends on the quiescence parameters (Figure 4b–d). The higher the rate of entry into the quiescent state (larger *α*) and the lower the rate of exit from the quiescent state (lower *β*), the higher the probability of treatment failure. In order to explain this, we will consider generating resistance to two drugs; higher numbers of drugs can be treated similarly. We build our arguments as follows.

#### The number of cycling 1-hit mutants is independent of the quiescence parameters

**Figure 6:**
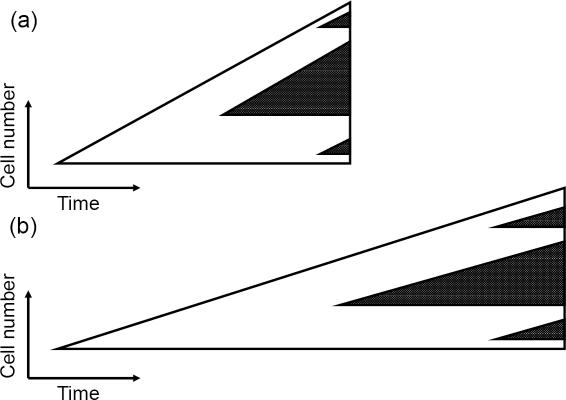
The expected number of one-hit mutants does not depend on the presence of quiescence. (a) represents a colony with no quiescence, and there is quiescence in (b). The white triangles depict growing colonies of cells (cells with quiescence grow slower). The end size is the same in both cases. Dark triangles represent growing mutant clones inside the colonies. The total number of mutant colonies is the same in both cases (the same number of cell divisions). The mutant colonies in (b) have a longer time to grow, but at the same time they grow slower. Therefore the resulting frequency of mutants is the same in (a) and (b).

Cycling mutants are produced by cycling wild-type cells and they grow according to the same law as the cells producing them. When *α* increases (or *β* decreases), the mutant clones grow more slowly because of quiescence, but at the same time they have more time to grow, see Figure 6. In other words, the changes in the mutant growth are completely compensated by the change in the time of growth. Therefore, we conclude that the number of cycling 1-hit mutants in a colony of a given size is also independent of quiescence.

#### The more quiescence there is in the colony, the larger is the total number of quiescent wild-type cells

This result is actually a consequence of a more general statement, that for each cell type (that is, cells resistant to 0, 1, 2 etc drugs), the number of quiescent cells divided by the number of cycling cells is given by *α/(l−α)* (see Text S1, Section 3.2). The particular fact that we will need is that, up to a small correction, the number of quiescent wild-type cells in a colony of size *N* is given by *αN/l*, whereas the number of cycling wild-type cells is given by *(1-α/l)N* (here we assume that the mutation rate is small compared to *1*, which is a safe bet).

#### The probability of two-drug treatment failure (due to resistance) increases with the quiescence rate

**Figure 7:**
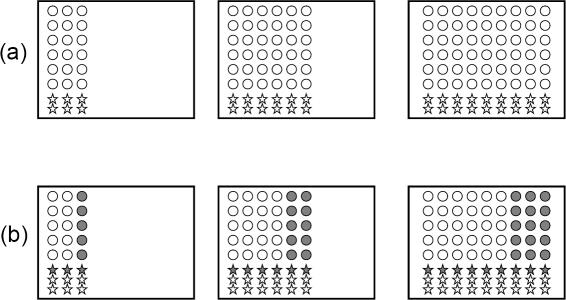
A schematic illustrating the argument stating that the probability to produce 2-hit mutants increases with quiescence. Each rectangle represents a colony of cells. There are three moments of time shown, first we have *N = 24*, then *N = 48* and finally *N = 72*. Circles represent wild-type cells, and stars–one-hit mutants. Gray shading denotes the state of quiescence for wild-type and mutant cells. In (a) we assume no quiescence (*α = 0*), whereas in (b) there is a probability to become quiescent (with α = *1/3)*. The number of cycling 1-hit mutants (empty stars) is the same in (a) and (b ) for the same values of *N*. The number of quiescent wild-type cells is given by the fraction α of all wild-type cells (e.g. 1/3 in (b)). At each moment of time, one of the cycling cells is picked for reproduction. We can see that the probability to pick a 1-hit mutant is always higher in (b) than in (a), because the fraction of cycling one-hit mutants increases as the tumor grows. Therefore, the probability to create a 2-hit mutant is higher in (b).

Our calculations show that the probability of treatment failure, caused by resistant mutants, rises with the level of quiescence in the context of therapy with two separate drugs (Figure 4b–d). This is a direct consequence of the previous two sections. Let us consider a colony consisting of wild-type and 1-hit mutant cells. Let us “watch” the colony grow by tracking each of *N-1* cell divisions, see Figure 7. Whenever a cell division happens, it may be a division of a cycling wild-type cell, or a division of a cycling 1-hit mutant cell. It is only the latter process which in principle may lead to the generation of two-drug resistance. The probability to create a double mutant at each division is proportional to the probability that a 1-hit mutant (and not a wild-type) cell divides. The number of cycling wild-type cells in a colony of a given size is a decreasing function of *α* , whereas the number of cycling 1-hit mutants is independent of *α* (see the two previous paragraphs). Therefore, as *α* increases, the relative abundance of cycling 1-hit mutants increases. In other words, among all cycling cells, the percentage of mutants increases with *α*, and so does the probability to create 2-hit mutants. Thus, the probability of resistance generation against 2 drugs increases with quiescence parameters.

#### Generalizations

These results can be generalized. First of all, we can show by similar methods that the probability of mutant generation increases with quiescence for 3- and higher-degree mutants (Figure 4). In fact, the dependence becomes stronger for larger numbers of drugs. However, we need to keep in mind that the actual probability of resistance becomes lower the more drugs we use, because it takes more mutation events to generate mutants simultaneously resistant to several drugs. Finally, all the results derived here apply for systems with a nonzero death rate, and a nonzero rate of cell “awakening”, *β*, see Text S1, Section 3.4.

### Cell death and mutant generation–a comparison

In a previous paper, we examined the effect of cell death on the probability of treatment failure as a result of acquired drug resistance [41]. We found a very similar pattern. The probability of treatment failure was independent of the death rate of tumor cells in the context of therapy with a single drug, which was also found in earlier studies by [43]. However, when treatment was assumed to occur with two or more drugs, the probability of treatment success depended on the death rate of tumor cells. The higher the death rate of tumor cells relative to their division rate, the higher the probability that mutant cells that are resistant against all drugs induce failure of therapy. While this result is identical to that observed for cellular quiescence, the reason for it is different. It is explained in the remaining part of this section.

#### The probability of pre-existence of one-hit resistant mutants is independent of the death rate

The probability of creating resistance before the start of treatment is defined by the probability to have at least one 1-hit mutant at a given colony size, which is given by *(probability to create a mutant) x (probability for a mutant clone to survive)*.The probability to create a mutant clone is proportional to the number of cell divisions. In turn, the number of cell divisions is a changing function of the death rate. With a zero death rate it takes exactly *N-1* cell divisions to go from *1* cell to *N* cells. As the death rate increases, it can take a lot more cell divisions to expand, because cell divisions are (partially) countered by cell deaths. Therefore, there are more cell divisions for a larger death rate, and as a consequence, more 1-hit mutants are produced. However, the probability for a mutant to survive is a decreasing function of the death rate, which exactly compensates the gain in the number of clones produced. Therefore, the probability to create resistance against 1 drug is independent of the death rate.

It is interesting to note that the number of one-hit mutants is a growing function of both the death rate and the senescence rate, but for different reasons. If we increase the death rate, the total number of cell divisions to reach size *N* will increase, and so will the number of mutants (but the average size of a clone size will remain the same). If we increase α, the total number of divisions will not change but the average clone size will grow, again leading to an increase in the total mutant number.

#### The probability of pre-existence of two-hit resistant mutants increases with the death rate

While the probability to have 1-hit mutants is independent of the death rate, the average number of 1-hit mutants that are produced and survive by the time the tumor size reaches size *N* is an increasing function of the death rate. The reason is as follows. The mutants are produced more often at higher death rates (because of the increased total number of cell divisions). Thus, more mutants are seeded to undergo clonal expansion. However, the size of the mutant clones is independent of the death rate (in the same manner as it was independent of the quiescence parameters, see Fig. 4). Therefore, the total amount of 1-hit mutants present at size N is an increasing function of the death rate. As a direct consequence of this, the probability to have 2-hit mutants at size N is also an increasing function of the death rate. This explains why the likelihood of 2-drug resistance is a growing function of cell death. This result can be extended to a larger number of drugs.

## Discussion

In this paper, we have examined the effect of cellular quiescence in CML cells on the kinetics of the treatment response, and on the chances that treatment fails because of the generation of drug resistant mutants. This was done in the context of targeted therapy using small molecule inhibitors. In accordance with experimental data [29], [30], we found a parameter region in which initiation of treatment results first in a fast rate of CML cell decline, followed by a second phase that is characterized by a slower rate of CML cell decline. This is simply the consequence of the quiescence dynamics. Note however, that this behavior is not expected to be universal, since the model predicts alternative patterns of cell decline in other parameter regions. The decline could occur in a single phase with a single exponential rate of decline, or the first phase of decline can be slower, followed by a faster phase (a reverse biphasic decline). Whether these patterns can be observed in experimental data requires the accumulation of more data sets that document CML dynamics during drug therapy. In the context of the biphasic decline that is also observed in data, parameter combinations determine when the switch occurs to the second and slower phase of treatment, and the expected time it takes to drive the tumor cells extinct. If it takes too long to drive the tumor cells extinct, the practical implication is that drug treatment fails to eliminate the tumor. Variations in quiescence parameters could determine whether CML relapses after prolonged treatment with imatinib, as observed in many cases [29]–[32], or whether relapse does not occur, as observed in a small subset of patients [33].

These notions add to previous theoretical work that examines the decline of CML cells during therapy [29], [30]. The paper by Michor et al [29], [30] explains the bi-phasic decline of CML cells by a hypothesized differential susceptibility of CML cell subpopulations to the drug imatinib. It is argued that differentiated cells are readily attacked by the drug, while cancer stem cells are not affected by treatment. The study by Roeder et al [29], [30] also uses mathematical arguments to address the bi-phasic decline of CML cells during treatment. Their models included elements of competition of cells in stem cell niches, and also invoked the concept of cellular quiescence to account for the bi-phasic pattern of cell decline. While the study by Roeder et al [29], [30] also includes the concept of cellular quiescence, our model is different in nature, examines different questions, and is therefore complentary. For example, our explanation of the two phases of CML decline (one mainly driven by the eradication of cycling cells, and the second one the awakening and death of quiescent cells) is very different from the explanation proposed by Roeder et al [29], [30]. Also, our paper examines the role of quiescence in drug resistance generation in cancer, which is not discussed in the papers by Roeder et al [29], [30].

Overall, the mathematical models that take into account cellular quiescence in tumor growth are based on earlier mathematical work. In a series of papers [44]–[46], Gyllenberg and Webb examined the role of cellular quiescence on the pattern of tumor growth. Using ordinary differential equation models, they suggested that basic Gompertzian tumor growth can be explained by a non-linear phenomenon that arises from an increased probability for cells to enter quiescence at larger tumor sizes [45]. These dynamics of tumor growth have also been studied in the context of more complex age and size structured population models [44], [46] that revealed more biologically interesting properties.

The second half of our paper investigates the effect of cellular quiescence on the evolutionary dynamics of mutants that are resistant against targeted drug therapies. In this respect, we found that in the context of treatment with a single drug, quiescence parameters do not influence the probability that drug resistant mutants contribute to treatment failure. On the other hand, if the cancer is treated with a combination of two or more drugs with different targets, then increased quiescence promotes treatment failure as a result of drug resistant mutants. However, while cellular quiescence increases the time until the cancer cells are reduced to low numbers or driven extinct, we find that this prolonged treatment phase is irrelevant for the generation of drug resistant mutants. Instead, if treatment fails because of the presence of drug resistant mutants, then they will have evolved during the tumor growth phase before treatment was initiated. Thus, strategies aimed at shortening the treatment phase, for example by activating quiescent cells, will not reduce the chances that treatment fails as a result of drug resistance. Similarly, if the tumor responds well to a given treatment regime, prolonged therapy to prevent relapse will not increase the chances of treatment failure as a result of drug resistance.

Our theoretical framework should be further validated in the context of clinical studies. We have already shown that our model can describe the observed bi-phasic decline of CML cells upon therapy. The data plotted in Figure 3 hint that apart from the bi-phasic decline, other patterns of CML dynamics during treatment may be observed in clinical data, as suggested by our model. This requires further investigation. Finally, it will be important to address our result that quiescence contributes to the evolution of drug resistance if patients are treated with two or more drugs in combination. Apart from imatinib other targeted drugs are becoming available for the treatment of CML [23], [24], [35]. According to our model, variation in the outcome of treatment could be explained by differences in the number of quiescent cells that have been generated during tumor growth. This could be addressed by examining the degree of cellular quiescence that is found in a tumor before the start of treatment. Perhaps an experimentally simpler strategy would be to perform *in vitro* experiments, in which a tumor cell population is allowed to grow towards a certain size, after which it is treated with a combination of two or more drugs. This could determine the fraction of experiments in which the tumor evolves resistance, and correlate this with the amount of cellular quiescence found in the cell culture.

## Materials and Methods

Here, we describe our general modeling approaches. Further mathematical details and calculations are found in the Supplementary Information ( Text S1).

### Stochastic modeling

In order to study the dynamics of a cell population with quiescence, we use a stochastic modeling approach. Namely, we formulate a continuous time, discrete state-space birth-death process (with or without mutations), where the rates of cell divisions and cell death are *l* and *d* respectively, and where cells enter the state of quiescence with a rate α and wake up from quiescence with a rate β. The resulting linear 2-dimensional Markov process corresponds to the exponential distribution of the timing of various elementary events (such as cell divisions, death etc).

### Combinatorial mutation network

We model the generation of resistance as mutation events. In order to acquire resistance to one drug, a cell must gain one mutational hit. Cells resistant to two drugs are double-hit mutants, etc. We assume that there is no cross-resistance in the system, such that each mutation event gives rise to resistance to one drug, and not to the other drugs. All the (partially and fully) resistant types can be placed on a combinatorial mutation network. The structure of the couplings between the equations is read off from such a network.

### Pre-treatment and treatment regimes

We assume that before treatment starts, all cells satisfy *l>d*, that is, the division rate is larger than their death rate. For our calculations, we also assume that all the mutants are neutral before the beginning of therapy. This assumption is not a necessity and the general model allows for positively- and negatively-selected mutants. We model the treatment phase by assuming that susceptible and partially-resistant mutants are killed by the drugs, such that their death rate is larger than their division rate. The opposite is true for the fully-resistant phenotype. By using standard methods, we write down the Kolmogorov forward equation for the probabilities. The coefficients in this equations (the rate constants of all the processes) are different depending on whether we consider the pre-treatment phase or the treatment phase. From this point, we proceed in two different ways, described in the following two sections.

### Equations for the averages

We formulate ordinary differential equations (ODEs) for the first moments (the expected numbers of cycling and quiescent cells) and study their behavior. This is done both in the absence of mutations (to study cancer development and treatment without resistance) and in the presence of mutations (to study cancer development and treatment in the face of emerging resistant mutants). The ODEs are linear with constant coefficients, and exact analytical solutions are possible. These solutions are not always transparent, especially in the case of multiple drug treatments. To understand the behavior, we find approximations for various modes of growth and decay, and study relevant limiting cases.

### Probability generating function

We also derive a partial differential equation (PDE) for the probability generating function. Probability generating function is used to study the probability of colony extinction, probability of treatment success, and the probability of having resistant mutants at a given colony size. All these quantities are obtained by solving the PDE by using the method of characteristics, because the PDE is of a transport type. The solutions are calculated numerically, for a subset of parameters values. Their behavior is also studied analytically by looking at the ODEs for the characteristics, and analyzing various limits of the exact solution, as well as their fixed points.

## Supporting Information

Text S1 (0.15 MB PDF)
