# Supplementary material for: Effect of Cellular Quiescence on the Success of Targeted CML Therapy
Source: PLoS One. 2007 Oct 3;2(10):e990. doi: 10.1371/journal.pone.0000990 (PMC1991595; doi:10.1371/journal.pone.0000990)
Supplement: Text S1 — (0.15 MB PDF) [file pone.0000990.s001.pdf]

Effect of cellular quiescence on the success of targeted CML therapy

Supplementary Materials

Natalia L. Komarova[1,2] and Dominik Wodarz[2,1]

[1] Department of Mathematics, and [2] Department of Ecology and Evolution, University of California, Irvine, CA 92697

1 Modeling shrinking populations with quiescence

Suppose that cells divide with rate l, die with rate d, become quiescent with rate α, and "wake up" from quiescence with rate β. We will refer to parameters α and β as quiescence parameters. Note that the "amount of quiescence" in the colony correlates positively with α and negatively with β.

Let us denote by i the number of cycling cells and by j the number of quiescent cells. The system's state is defined by the pair (i, j). The dynamics of cells can be described by a following continuous time birth-death process. In an infenitesimally small interval of time, Δt, only the following changes in the system are possible:

- $(i, j) \to (i + 1, j)$ with probability $il\Delta t$, a cell division;
- $(i, j) \to (i - 1, j)$ with probability $id\Delta t$, a cell death;
- $(i, j) \to (i - 1, j + 1)$ with probability $i\alpha\Delta t$, a cell turning quiescent;
- $(i, j) \to (i + 1, j - 1)$ with probability $j\beta\Delta t$, a quiescent cell waking up;
- $(i, j) \to (i, j)$ with probability $1 - (i[l + d + \alpha] + j\beta)\Delta t$, no change.

The Kolmogorov forward equation for this system is:

$$\dot{\varphi}_{ij} = l(i-1)\varphi_{i-1} + d(i+1)\varphi_{i+1} + \alpha(i+1)\varphi_{i+1,j-1} + \beta(j+1)\varphi_{i-1,j+1} - \varphi_{ij}[(l+d+\alpha)i + \beta j]. \quad (1)$$

The initial condition is such that the number of cycling cells is given by I_0 and the number of quiescent cells is given by J_0 at time $t = 0$, i.e. $\varphi_{I_0,J_0}(0) = 1$, and $\varphi_{ij}(0) = 0$ for all other values of i and j.

1.1 Equations for the averages

Let us denote by x the average number of cycling cells, and y the average number of quiescent cells:

$$x = \sum_{i,j=1}^{\infty} \varphi_{ij} i, \quad y = \sum_{i,j=1}^{\infty} \varphi_{ij} j. \quad (2)$$

The equations for x and y are as follows:

$$\dot{x} = (l-d-\alpha)x+\beta y, \quad (3)$$
$$\dot{y} = \alpha x-\beta y, \quad (4)$$
$$x(0) = I_0, \quad y(0)=J_0. \quad (5)$$

This linear system of ODEs can be solved analytically. In particular, the eigenvalues of the corresponding matrix are:

$$\lambda_\pm = \frac{1}{2}\left((l-\alpha-d-\beta)\pm\sqrt{(d-l+\alpha+\beta)^2-4\beta(d-l)}\right)\leq 0, \quad (6)$$

such that

$$|\lambda_+|\leq|\lambda_-|.$$

The total number of cells, $n_{tot}(t)=x(t)+y(t)$, as a function time, is given by

$$n_{tot}=g_+e^{\lambda_+t}+g_-e^{\lambda_-t}, \quad (7)$$

with the constants

$$g_+=\frac{-\lambda_-}{\lambda_+-\lambda_-}\left[I_0\left(\frac{\lambda_+}{\beta}+1\right)+J_0\right], \quad g_-=\frac{\lambda_+}{\lambda_+-\lambda_-}\left[I_0\left(\frac{\lambda_-}{\beta}+1\right)+J_0\right].$$

The function $\log n_{tot}(t)$ has two asymptotes: $\log n_{tot}\to\log g_-+\lambda_-t$ as $t\to$-$infty$, and $\log n_{tot}\to\log g_++\lambda_+t$ as $t\to+\infty$, see figure 1. It is possible to show that $g_+\geq 0$. There are three cases: (a) $g_->g_+$, (b) $0\leq g_-\leq g_+$ and (c) $g_-<0$. If condition (a) is satisfied, then the two asymptotes intersect at a positive t, and we have a biphasic decline (see below), where an initial fast phase of decay is followed by a slow phase. If (b) holds, there is essentially one wave of decline (the first wave is not pronounced). Finally, if (c) holds, we have a "reversed biphasic decline", where the function $\log n_{tot}$ decays slower at first and then faster.

1.2 Biphasic decline

If the following inequality holds[1]:

$$\frac{J_0}{I_0}\ll\frac{d-l-\alpha-\beta}{d-l+\alpha+\beta},$$

then the function $n_{tot}(t)$ has a characteristic shape of a "biphasic decline". There are two waves of decay, first the fast one at the rate λ_-, and then the slower one, at the rate λ_+. Note that if $\alpha\ll(d+\beta-l)$, the two eigenvalues are $-\beta$ and $-(d-l)$. The time of switching from the first to the second phase is obtained from the equation $g_-e^{\lambda_-t}=g_+e^{\lambda+-t}$, such that

$$T_{switch}=\frac{\log\left(\frac{g_-}{g_+}\right)}{\lambda_+-\lambda_-}.$$

[1]This is equivalent to $g_+/g_-\ll 1$.

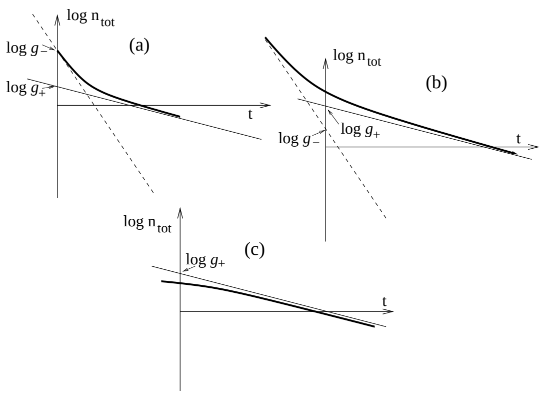

Figure 1: The three types of population decline: (a) Biphasic decline, (b) One phase of decline and (c) Reverse biphasic decline. The thick line represents the function $\log n_{tot}$, the thin solid line is $\log g_+ + \lambda_+ t$, and the dashed line is $\log g_- + \lambda_- t$. In (c), the dashed line is absent because $g_- < 0$.

In the case where $J_0 \ll I_0$, we have

$$T_{switch} = \frac{\log \left\{ \frac{d-l-\alpha-\beta+\sqrt{(l-\alpha+\beta+d)^2-4\beta(d-l)}}{-d+l+\alpha+\beta+\sqrt{(l-\alpha+\beta+d)^2-4\beta(d-l)}} \right\}}{\sqrt{(l-\alpha+\beta+d)^2-4\beta(d-l)}}.$$

In particular, if α is small, we have the following simplified expression:

$$T_{switch} = -\frac{\log \frac{(d-l)\alpha}{(d-l-\beta)^2}}{d-l-\beta}.$$

1.3 The time until extinction

The time it takes to reduce the total population to small numbers can be estimated by solving the equation $x(t) + y(t) = 1$ for t. Approximately, this is given by $g_+ e^{\lambda_+ t} = 1$, or

$$T_{treat} = \frac{\log g_+}{|\lambda_+|}. \tag{8}$$

A similar expression for the time of treatment is obtained from studying the probability of extinction, $\varphi_{0,0}(t)$, equation (1).

1.4 Fitting the model with the data

We have fitted out model with the experimental data of [4] and [6]. The data shows the relative number of CML cells in patient's blood. More precisely, [4] plots the ratio of BCR-ABL transcripts to BCR transcripts, while [6] shows the ratio of BCR-ABL transcripts to ABL transcripts in peripheral blood of patients treated with Imatinib.

Formula (7) was used to fit the data. Although the formula was derived for the absolute number of cells, it is capable of measuring the ratios, as long as the "base" measurement remains constant. Thus it is assumed that in the experiments, the number of non-leukemic cells remains roughly constant throughout the experiment.

We used a standard least-square algorithm to approximate the logarithm of the data with a piecewise-linear function representing the two stages of decline. To simplify the fitting procedure, we used the assumption that $\alpha \ll (d+\beta-l)$. In this case the first slope yields the value of $d-l$, and the second slope yields the parameter β. Next, we had to extract the value of the two remaining parameters, α and J_0 (the value of I_0 is obtained from $I_0 = N_0 - J_0$, where N_0 is the measurement at time zero). As α decreases, the error of the fit first decreases and then flattens out and stays essentially constant (and small) for small values of α. The corresponding values of J_0 grow as α decays, but also saturate at a certain low level. This suggests that the shape of the curve is not sensitive to the exact value of α, as long as it is sufficiently small.

These observations make sense because smaller values of α (a smaller production rate of quiescent cells) can be somewhat compensated by higher initial numbers of quiescent cells. Moreover, even though in formula (7), the values of α and J_0 are independent parameters, in reality they are strongly linked. As the colony grows, the amount of quiescent cells at size N, J_0, is defined by the rate of quiescence, α, according to

$$J_0/N_0 \approx \alpha/l,$$

see formula (20) below. Thus in order to correctly determine the parameter α, we would need to use the growth-phase of the cell colonies.

To conclude, we note that the least-square procedure yields estimates for the decay rate of cells, $d-l$, the rate of awakening, β, and the ranges for α and J_0. These calculations demonstrate that the model is capable of capturing the behavior of CML cells treated by Imatinib, and the parameters of the biphasic decay can be determined from the quiescence rates. However, the analysis presented here is only an illustration of the method. The fitting procedure should be repeated for future data sets which show the mean rather than the median, and also include information on the absolute numbers of non-CML cells.

2 Modeling resistance to drugs in a population with quiescence

Here we include the possibility of mutations in the birth-death-quiescence process. This is an extension of the framework of [1, 2], where generation of resistance by cells *without quiescence* was studied.

2.1 Stochastic model

A cell can be either resistant or susceptible to each of the m drugs. In total, there are $2^m - 1$ resistant types. They can be enumerated by a binary index s, which is a string of m components with numerical values from the set $\{0, 1\}$.

We denote by i_s the number of cycling cells of resistance type s, and j_s the number of quiescent cells of resistance type s. Each type s is characterized by its division rate, l_s, and death rate, d_s. Also, all the types are naturally placed on a binary directed network where each edge connects two types which only differ by their resistance properties with respect to one drug, see [1]. A single mutation separates each pair of connected types. The Kolmogorov forward equation for this system can be written (not shown). The following probability generating function can be defined:

$$\Psi(\xi_0, \eta_0, \dots, \xi_n, \eta_n; t) = \sum_{s=0}^{n} \varphi_{i_0, j_0, \dots, i_n, j_n} \xi_0^{i_0} \eta_0^{j_0} \dots \xi_n^{i_n} \eta_n^{j_n}$$

(the expression above can be treated as a transformation from discrete variables $i_0, j_0, \dots i_n, j_n$ to continuous variables $\xi_0, \eta_0, \dots, \xi_n, \eta_n$). This function satisfies the PDE (see e.g. [5], [7], Chapter 3; [2]):

$$\frac{\partial \Psi}{\partial t} = \sum_{s=0}^{n} \left(\frac{\partial \Psi}{\partial \xi_s} \left(l_s (1 - \sum_j u_{s \to j}^{out}) \xi_s^2 + \sum_j l_s u_{s \to j}^{out} \xi_j^{out} + d_s + \alpha_s \eta_s - (l_s + d_s + \alpha_s) \xi_s \right) - \frac{\partial \Psi}{\partial \eta_s} \beta_s \eta_s \right).$$

For each type s, the quantities $u_{s \to j}$ are the mutation rates corresponding to the transformations of type s into each of the types j, with which s is directly connected. The types j are assumed to be downstream from type s. The linear transport-type PDE can be solved by the standard method of characteristics:

$$\dot{\xi}_s = l_s (1 - \sum_j u_{s \to j}^{out}) \xi_s^2 + \sum_j l_s u_{s \to j}^{out} \xi_j^{out} + d_s + \alpha_s \eta_s - (l_s + d_s + \alpha_s) \xi_s, \quad (9)$$

$$\dot{\eta}_s = -\beta_s \eta_s. \quad (10)$$

If initially we have M_0 cycling cells of resistance type 0, then we have

$$\Psi(\xi_0, \eta_0, \dots, \xi_n, \eta_n; 0) = \xi_0^{M_0}.$$

To find the function $\Psi(\bar{\xi}_0, \bar{\eta}_0, \dots, \bar{\xi}_n, \bar{\eta}_n; \bar{t})$, we need to solve the characteristics equations with the initial conditions

$$\xi_s(0) = \bar{\xi}_s, \quad \eta_s(0) = \bar{\eta}_s,$$

and obtain

$$\Psi(\bar{\xi}_0, \bar{\eta}_0, \dots, \bar{\xi}_n, \bar{\eta}_n, \bar{t}) = [\xi_0(\bar{t})]^{M_0}.$$

All the resistant types can be separated into classes such that in each class k, all the types are resistant to exactly k drugs (and susceptible to $m - k$ drugs).

Let us suppose that within each class, the birth and death rates are equal, and also that all mutation rates are equal to each other. Then the equations for all types within the same class are identical, and we have for each class k:

$$\dot{\xi}_k = l_k(1-(m-k)u)\xi_k^2 + [(m-k)l_k u\xi_{k+1} - (l_s + d_k + \alpha_k)]\xi_k + d_k + \alpha_k\eta_k, \quad (11)$$

$$\dot{\eta}_k = \beta_k(\xi_k - \eta_k), \quad 0 \le k \le m. \quad (12)$$

The coefficients in equations (11-12) depend on whether treatment takes place or not. We assume that before the start of treatment, the growth and death rates of all types are the same:

$$l_k = l, \quad d_k = d < l, \quad 0 \le k \le m.$$

After the treatment starts, we assume that the fully-susceptible and all the partially resistant types are killed by the drugs with the intensity H, and the fully-resistant type is not affected by treatment. This translates into the following values for the coefficients:

$$l_k = l \quad \forall k, \quad d_k = d + H > l, \quad 0 \le k \le m-1, \quad d_m = d.$$

2.2 Probability of resistance generation during treatment

Suppose that at the start of treatment we have I_0 cells of class 0, …, I_m cells of class m. Then the probability of treatment success is given by

$$P(treat.\ succ.) = \lim_{t\to\infty} \Psi(0,\dots,0;t) = \prod_{k=0}^{m} [\xi^\infty]^{I_k},$$

where $\xi_k^\infty = \lim_{t\to\infty} \xi_k(t)$. From studying the fixed points of system (11-12), we can see that the limiting values ξ_k^∞ do not depend on the quiescence parameters α and β. This means that the probability of treatment success (for a given colony) is not affected by the process of quiescence.

2.3 The probability of resistance generation before treatment.

The probability to not have a single resistant mutant at time t_N is given by $[\xi_0(t_N)]^{M_0}$, where $\xi_0(t)$ is a component of the solution of system (9-10) with the initial conditions,

$$\xi_0(t) = \ldots = \xi_{m-1} = 1, \quad \xi_m = 0.$$

The probability of not having resistance in a colony of size N can be estimated as follows. We assume that the size N of the colony and the time t are connected by the deterministic relation, $x(t_N) + y(t_N) = N$, where, as before, $x(t)$ is the expected total number of cycling cells (of all types), and $y(t)$ is the expected total number of quiescent cells (the validity of this assumption is discussed in [3]). The functions $x(t)$ and $y(t)$ satisfy the system (3-4) with the initial condition $x(0) = M_0$, $y(0) = 0$.

The probability of not having resistance in a colony of size N is then given by

$$Prob(no\ resistance\ at\ size\ N) = \frac{[\xi_0(t_N)]^{M_0} - (d/l)^{M_0}}{1 - (d/l)^{M_0}}.$$

This follows from the fact that the probability of having no resistant mutants ($[\xi_0(t_N)]^{M_0}$) equals the probability of going extinct before reaching size N ($(d/l)^{M_0}$), plus the probability to grow to size N and to have no resistant mutants ($Prob(no\ resistance\ at\ size\ N)$).

3 The average number of mutants at size N

Another way to study the production of resistant mutants is to look at the expected behavior (the first moments) of various types of cells. We can derive equations for the moments from the Kolmogorov forward equations.

3.1 Equations for the moments

To gain intuitive understanding of the dependence of mutant production upon quiescence, we consider a simplified system for m drugs with $d = 0$:

$$\dot{x}_0 = [l(1-u_0) - \alpha]x_0 + \beta y_0, \tag{13}$$

$$\dot{y}_0 = \alpha x_0 - \beta y_0, \tag{14}$$

$$\ldots \tag{15}$$

$$\dot{x}_k = l u_{k-1} x_{k-1} + [l(1-u_k) - \alpha]x_k + \beta y_k, \tag{16}$$

$$\dot{y}_k = \alpha x_k - \beta y_k, \quad 0 < k \leq m. \tag{17}$$

$$\tag{18}$$

Here, the rates u_k are probabilities of mutation from class k to class $k+1$. Because of the combinatorial nature of our problem, we have

$$u_k = (m-k)u,$$

where u is a basic rate of mutations.

3.2 The expected number of mutants for $\beta = 0$

Let us assume that $\beta = 0$. Then linear system (13-17) can be solved analytically (the simplifying assumption that $\beta = 0$ makes the equations for cycling cells independent of the equations for quiescent cells). In particular, for any value of k, we have the exact solution

$$\sum_{k=0}^{m}(x_k + y_k) = \frac{l e^{(l-\alpha)t} - \alpha}{l - \alpha}.$$

Therefore, the time it takes to reach size N is given by

$$t_N = \frac{\log\left[\frac{N(l-\alpha)+\alpha}{l}\right]}{l-\alpha}. \quad (19)$$

To calculate the fraction of cycling cells of class k at size N, we substitute the above expression for t_N into the exact solution for $x_m(t)+y_m(t)$, and expand in a double Taylor series in terms of small parameters u and $1/N$. We obtain:

$$\frac{x_k}{x_k+y_k} = \left(1-\frac{\alpha}{l}\right)(1+O(1/\log N)) + O(u^{-1}). \quad (20)$$

Similarly, the average number of mutants resistant to m drugs is given by

$$x_m(t_N)+y_m(t_N) \approx N\left(\frac{u\log N}{1-\frac{\alpha}{l}}\right)^m. \quad (21)$$

Again, here we only present the first term in the u-expansion and the largest term in the $1/\log N$ expansion of the exact expressions.[2] Exact values of quantities $x_k(t_N)+y_k(t_N)$ and $\frac{x_k}{x_k+y_k}$ are plotted in figure 2(a,b) as functions of α.

Let us first consider the case $m=1$. The average number of (cycling and quiescent) 1-hit mutants in this case is an increasing function of α, see equation (21). On the other hand, the average number of *cycling* 1-hit mutants is obtained from equations (21) and (20) and is given by $Nu\log N$, that is, it is independent of α. To understand this result, we note that cycling mutants are produced by cycling wild-type cells and they grow according to the same law as the cells producing them. When α increases, the mutant clones grow more slowly because of quiescence, but at the same time they have more time to grow (it takes more "events" before the number of cells reaches size N). In other words, the changes in the mutant growth are completely compensated by the change in the time of growth. That is why the quantity $x_1(t_N)$ is a function of the colony size only, see figure 2(c).

The dependence of the total number of mutants, $x_m(t_N)+y_m(t_N)$, on α becomes stronger as the number of drugs increases, see figure 2. In particular, both the total number of 2-hit mutants and the number of cycling 2-hit mutants depend on α, formula (21).

3.3 The probability of having resistance to m drugs, $\beta = 0$

In our studies of resistance generation, two quantities are of particular importance: the expected number of resistant mutants, and the probability of having resistance.

Of course, the latter quantity (the probability of having resistance) cannot be obtained directly when studying system (13-17). However, we can study a

[2]In order to perform the Taylor series expansion we assume that $u \ll 1-\alpha/l$; for α very close to l, the value $1-\alpha/l$ would have to be taken to be the small parameter of expansion.

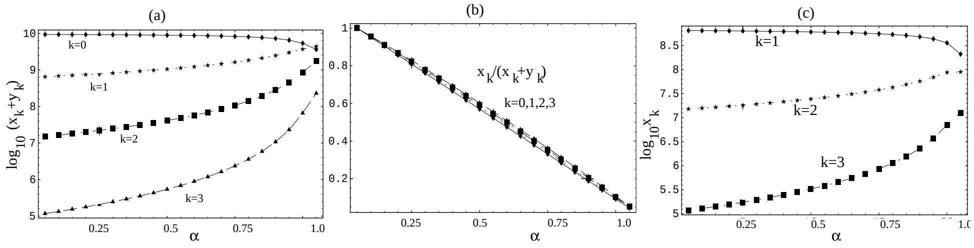

Figure 2: A numerical illustration of the dependence of solutions of (13-17) on α, with $\beta = 0$. (a) The total number of mutants of class k, $x_k + y_k$; (b) The fraction of cycling mutants of class k, $\frac{x_k}{x_k+y_k}$; (c) The number of cycling mutants of class k, x_k. The parameters are $n = 10^10$, $m = 3$, $l = 1$, $u = 10^{-3}$.

related quantity is $W_{m-1} \equiv \int_0^{t_N} lux_k(t)\,dt$, the total number of fully resistant mutants produced by the colony. We have

$$W_{m-1} = mNu\left(\frac{u\log n}{1-\frac{\alpha}{l}}\right)^{m-1}. \tag{22}$$

In particular, for $m = 1$, both W_0 and the probability of generating resistance to one drug is independent of α. However, the dependence on α does take place for larger values of m. For example, the probability of resistance to 2 drugs is an increasing function of α.

The case of $m = 2$ drugs can be explained intuitively. Two-hit mutants are produced by cycling one-hit mutants. At each value of N, the number of cycling wild-type cells is given by

$$x_0(t_N) = (1-\alpha/l)N(1+o(u)),$$

that is, it decreases with α, and the number of cycling one-hit mutants is

$$x_1(t_N) = mNu\log N(1+o(u)),$$

which is α-independent (the last two expressions are obtained by a direct solution of system (13-17) and using equation (19)). We can see that the relative proportion of one-hit mutants for a given colony size is a growing function of α. Therefore, for increasing α, every time a cell divides, it becomes more likely that the dividing cell already has a mutation. Therefore, the production of two-hit mutants is a growing function of α, see the expression for W_1, equation (22).

3.4 The case $\beta > 0$

Once we include a nonzero value of β in system (13-17), the equation $\sum_{k=0}^{m}(x_k + y_k) = N$ cannot be analytically resolved for t_N. In other words, there is no equivalent of formula (19) with $\beta > 0$. In order to see how the results depend on the value of β, we have performed numerical solutions of system (13-17). These are presented in figure 3. We can see that the total number of mutants

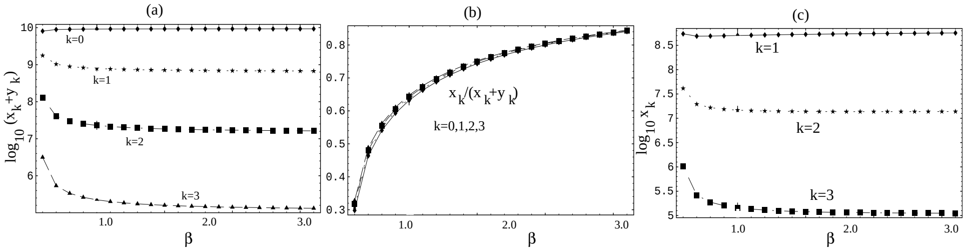

Figure 3: The dependence of solutions of (13-17) on β. (a) The total number of mutants of class k, $x_k + y_k$; (b) The fraction of cycling mutants of class k, $\frac{x_k}{x_k+y_k}$; (c) The number of cycling mutants of class k, x_k. The parameters are as in figure 2, with $\alpha = 0.7$.

of class k decays with β (figure 3(a)), and the fraction of cycling cells increases with β (figure 3(b)). The number of cycling mutants, x_k, is a decaying function of β for $k > 1$ (figure 3(c)).

These results show that the dependence of the number of resistant mutants on the rate of cell awakening, β, is the opposite of that on the rate of quiescence, α. In general, the number of resistant mutants increases with the amount of quiescence in the system. The probability of resistance to k drugs (which is related to the function x_{k-1}, formula (22)), also increases with the amount of quiescence, except for the case $k = 1$, where it is independent of the amount of quiescence. This illustrates the results of stochastic calculations of Section 2 (see figure 2 of the main paper) at an intuitive level.

References

[1] Komarova, N.L. and Wodarz, D. (2005) Drug resistance in cancer: principles of emergence and prevention. Proc. Natl. Acad. Sci. 102 (27), pp. 9714-9.

[2] Komarova, N.L. (2006) Stochastic modeling of drug resistance in cancer. Jour. Theor. Biol. , 239(3), pp. 351-366.

[3] Komarova, N.L., Baldi, P. and Wu, L. (2007) The fixed-size Luria-Delbruck model with a nonzero death rate. Math. Biosciences doi:10.1016/j.mbs.2007.04.007

[4] , Michor, F., Hughes, T.P., Iwasa, Y., Branford, S., Shah, N.P., Sawyers, C.L. and Nowak, M.A. (2005) Dynamics of chronic myeloid leukaemia, Nature 435(7046), pp. 1267–1270.

[5] Moolgavkar, S.H., Dewanji, A. and Venzon, D.J. (1988) A stochastic two-stage model for cancer risk assessment. I. The hazard function and the probability of tumor. Risk Anal. 8(3), pp. 383–92.

[6] Roeder, I., Horn, M., Glauche, I., Hochhaus, A., Mueller, M. C. and Loeffler, M. (2006) Dynamic modeling of imatinib-treated chronic myeloid leukemia: functional insights and clinical implications, Nat Med 12(10), pp. 1181–1184.

[7] Wodarz, D. and Komarova, N.L. (2005) *Computational Biology of Cancer: Lecture notes and mathematical modeling.* World Scientific Publishing.
